# Evaluation of MTT Heterogeneity of Perfusion CT Imaging in the Early Brain Injury Phase: An Insight into aSAH Pathopysiology

**DOI:** 10.3390/brainsci13050824

**Published:** 2023-05-19

**Authors:** Björn B. Hofmann, Igor Fischer, Daniel M. Donaldson, Yousef Abusabha, Cihat Karadag, Sajjad Muhammad, Kerim Beseoglu, Daniel Hänggi, Bernd Turowski, Christian Rubbert, Jan F. Cornelius, Marcel A. Kamp

**Affiliations:** 1 Department of Neurosurgery, Medical Faculty and University Hospital Düsseldorf, Heinrich-Heine-University Düsseldorf, 40225 Düsseldorf, Germany; 2 Department of Neurosurgery, International Neuroscience Institute, 30625 Hannover, Germany; 3 Department of Diagnostic and Interventional Radiology, Medical Faculty and University Hospital Düsseldorf, Heinrich-Heine-University Düsseldorf, 40225 Düsseldorf, Germany; 4 Centre for Palliative and Neuro-Palliative Care, Brandenburg Medical School Theodor Fontane, Campus Rüdersdorf, 15562 Rüdersdorf bei Berlin, Germany; 5 Faculty of Health Sciences Brandenburg, Brandenburg Medical School Theodor Fontane, 16816 Neuruppin, Germany

**Keywords:** aneurysmal subarachnoid hemorrhage, early perfusion, computed tomography (CT), MTT heterogeneity, microvascular perfusion heterogeneity, capillary transit time heterogeneity, mean transit time, global transient ischemia, early brain injury

## Abstract

The concept of early brain injury (EBI) is based on the assumption of a global reduction in brain perfusion following aneurysmal subarachnoid hemorrhage (aSAH). However, the heterogeneity of computed tomography perfusion (CTP) imaging in EBI has not yet been investigated. In contrast, increased mean transit time (MTT) heterogeneity, a possible marker of microvascular perfusion heterogeneity, in the delayed cerebral ischemia (DCI) phase has recently been associated with a poor neurological outcome after aSAH. Therefore, in this study, we investigated whether the heterogeneity of early CTP imaging in the EBI phase is an independent predictor of the neurological outcome after aSAH. We retrospectively analyzed the heterogeneity of the MTT using the coefficient of variation (cvMTT) in early CTP scans (within 24 h after ictus) of 124 aSAH patients. Both linear and logistic regression were used to model the mRS outcome, which were treated as numerical and dichotomized values, respectively. Linear regression was used to investigate the linear dependency between the variables. No significant difference in cvMTT between the patients with and those without EVD could be observed (*p* = 0.69). We found no correlation between cvMTT in early CTP imaging and initial modified Fisher (*p* = 0.07) and WFNS grades (*p* = 0.23). The cvMTT in early perfusion imaging did not correlate significantly with the 6-month mRS for the entire study population (*p* = 0.15) or for any of the subgroups (without EVD: *p* = 0.21; with EVD: *p* = 0.3). In conclusion, microvascular perfusion heterogeneity, assessed by the heterogeneity of MTT in early CTP imaging, does not appear to be an independent predictor of the neurological outcome 6 months after aSAH.

## 1. Introduction

Aneurysmal subarachnoid hemorrhage (aSAH) remains a disease with a potentially devastating outcome [1]. The pathophysiological sequelae following aSAH can be simplistically divided into the phase of early brain injury (EBI, first 72 h after ictus) and a second phase, named delayed cerebral ischemia (DCI), in the later phase after ictus [2,3,4,5,6]. Various clinical and translational studies suggest different underlying pathomechanisms for the different stages of the disease.

The concept of EBI has gained increasing attention in recent years [6,7,8,9]. The bleeding event results in a sudden peak in intracranial pressure (ICP, as high as the mean arterial pressure within one minute of ictus), with consequent transient global ischemia with a steeply decreased cerebral blood flow (CBF) and volume (CBV), sometimes resulting in a temporary intracranial circulatory arrest [10,11]. Various methods are available for assessing cerebral perfusion, including dynamic susceptibility contrast MRI and contrast-enhanced near-infrared spectroscopy, which have broad applications in different pathologies [12,13,14]. However, for patients with aSAH, CTP imaging has become the clinically established choice, due to its rapid availability and quick operability. Clinically, early cerebral perfusion impairment, assessed by computed tomography perfusion (CTP)-based MTT impairment, is a predictor of the later occurrence of DCI and an unfavorable outcome [7,8,15]. Early CTP scanning is, therefore, a valuable tool to identify patients who are at risk for DCI and allows us to prompt more robust preventative measures and treatment in this subgroup [7,15].

Not only in EBI, but also in the DCI phase, perfusion imaging protocols have been used to study the underlying pathophysiology and have been implemented in the clinical routine of some neurovascular centers [16,17]. However, perfusion protocols and post-processing are heterogeneous, and the results in the literature regarding prognosis prediction are not conclusive [18]. Yet, the surrogate marker mean transit time (MTT) of CTP imaging in particular is a sensitive tool used to detect DCI-related perfusion impairment that is used in the clinical management of aSAH patients and considered a predictor of an unfavorable outcome [7,19,20].

In addition, the restriction of general microvascular perfusion itself is probably not the only decisive factor in the pathomechanism of DCI. The heterogeneity of microvascular perfusion is probably also a feature of DCI. Østergaard and colleagues hypothesized that a high capillary transit time heterogeneity (CTH), in addition to the impairment of cerebral perfusion itself, is a reason for a DCI-related poor outcome [21,22,23,24]. High CTH describes inhomogeneous microvascular blood distribution across the capillaries, resulting in decreased net tissue oxygenation of brain tissue in the affected area, for instance, via a shunt effect [21,22,24]. Recently, we were able to link a new CTP imaging parameter depicting microvascular perfusion heterogeneity in the DCI phase to the outcome of patients after aSAH. We demonstrated that the heterogeneity of MTT in the DCI phase, 72 h–21 days post ictus, significantly correlates with the 6-month outcome in patients with aSAH [25]. A low coefficient of variation of MTT (cvMTT), a measure of the relative standard deviation of MTT, significantly correlates with a favorable 6-month mRS in patients with aSAH [8].

However, the heterogeneity of microvascular perfusion impairment, which is defined as the heterogeneity of the MTT in the EBI phase, has not yet been investigated. In the present study, we analyzed the heterogeneity of MTT in early perfusion CT imaging after aSAH as a potential independent predictor of neurological outcome.

## 2. Materials and Methods

### 2.1. Ethical Statement

The retrospectively evaluated data originate from a monocentric, prospective observational study conducted between January 2008 and December 2015. All procedures performed in the studies involving human participants were in accordance with the ethical standards of the institutional committee and the 1964 Helsinki declaration and its later amendments. The local ethics committee of the Medical Faculty of the Heinrich-Heine University, Düsseldorf, Germany, approved this study (study ID: 5760R). We prepared the manuscript according to the Strengthening the Reporting of Observational Studies in Epidemiology (STROBE) guidelines.

### 2.2. Inclusion Criteria

All patients with SAH admitted to our neurovascular center were retrospectively selected for this study according to the following inclusion criteria: (1) aneurysmal SAH documented by an initial non-enhanced CT, as well as CT and/or digital subtraction angiography; (2) CTP imaging within 24 h after ictus (determined by a self-report of the headache event or a third-party report of sudden neurological deterioration or loss of consciousness. If a prolonged headache was present or the time of bleeding could not be determined with certainty, patients were excluded from the analysis); and (3) a documented clinical follow-up 6 months after discharge to assess the functional outcome. Patients were excluded from this study based on the following criteria: (1) patients with unevaluable CTP (e.g., due to severe motion), (2) patients with pre-treated aneurysms or recurrent aSAH, and (3) patients with a history of previous cranial neurosurgical or endovascular procedures.

### 2.3. aSAH Management

If an in-house non-enhanced CT scan showed evidence of aSAH, supplemental perfusion imaging was immediately performed in the same session. The patients who had a CT scan in a peripheral hospital with evidence of aSAH were transferred to our neurovascular center as soon as possible and received an immediate CTP scan upon arrival at our hospital. The treatment of aSAH patients in the early phase after bleeding was based on an in-house guideline following the current aSAH guidelines [26]. Systolic blood pressure was maintained between 100 and 140 mmHg before aneurysm occlusion. All patients with a GCS of ≤12 (patients with a poor WFNS grade) were intubated and ventilated before aneurysm repair, and the partial pressure of carbon dioxide (pCO_2_) was maintained between 30 and 35 mmHg. External ventricular drainage (EVD) was established in the intubated patients and the patients with a poor WFNS grade, as well as in the patients with exacerbated intracranial pressure after initial imaging.

### 2.4. Perfusion CT Analysis

CTP scanning was performed as previously described [25], as follows: CTP data were acquired using a multi-slice CT scanner (SOMATOM Volume Zoom, Definition Flash or AS, Siemens Erlangen, Germany, 80 kV, 120 mAs, two adjacent slices, 10 mm slice thickness, 1 image/s over 35 s) [7,8,20,25]. Three seconds after starting the CT scan, contrast-enhancing agents (30 mL, 400 mg iodine/mL), followed by a saline chaser (30 mL), were injected at a flow rate of 5 mL/s. Intravenous access of ≤ 18 gauge into a cubital vein or a high-flow central venous catheter was required for contrast administration. The slices were positioned at the level of the central portions of the lateral ventricles, parallel to a plane through the orbital floor and external auditory meatus, thereby capturing the areas of the anterior, middle, and posterior cerebral artery territories, as well as the anterior and posterior marginal zones [25,27]. Parameter images (MTT, CBF, CBV, and Tmax) were calculated using STROKETOOL-CT (Digital Image Solutions, Frechen, Germany), which processed the data using singular value decomposition (SVD) [25,28]. We used Angiotux CT 2D software (ECCET 2006, Beck A. Aurich V.) for standardized parameter value extraction from the different cortical brain regions [20,25]. Both acquired slices were manually reviewed to select the slice that best matched the above criteria in order to best capture the supratentorial arterial territories. A 10-mm-wide band was automatically delineated along the cortex, omitting the outer cerebrospinal fluid spaces, the rostral falx cerebri, and the superior sagittal sinus. The automated region of interest (ROI) definition was superimposed on the respective slice for review. Potential deviations from the ideal shape were manually corrected as needed. Then, a running average spanning 10° of the ROI was computed in 2° steps for each perfusion parameter, yielding 180 measurements per parameter per CTP scan.

### 2.5. Definition of Outcome Measures

We defined the heterogeneity of microvascular perfusion as the heterogeneity of cerebral perfusion among the measured ROIs and assessed the heterogeneity of microvascular perfusion using the coefficient of variation (cv or relative standard deviation) of the MTT in a single slice for each scan. The cv is a measure of the dispersion of a probability distribution independent of the mean value of the examined variable [25], as follows:

$$cvMTT=\frac{standard\;deviation}{expected\;value}=\frac{standard\;deviation\;MTT}{mean\;MTT}$$

A schematic representation of the workflow is provided in the Supplementary Materials. If a patient received more than one CTP scan within 24 h after ictus, only the first imaging was included in the analysis of this study.

The initial neurological condition at admission to our department was dichotomized using the World Federation of Neurologic Surgeons (WFNS) grading system [29], as follows: A WFNS score of 1–3 represents a good neurological grade upon admission, whereas a WFNS score of 4–5 is considered a poor grade. The modified Rankin scale (mRS) was used to evaluate the functional outcome six months after ictus [30]. An mRS score of 0–2 was considered a favorable outcome, while a score of 3–6 was considered an unfavorable outcome.

### 2.6. Statistical Analysis

Student’s *t*-test was used to compare the numerical values between two groups. Linear dependency between ordinal variables (Fisher score, WFNS grade) and a continuous variable (cvMTT) was modeled by treating the ordinal variable as numerical and performing linear regression. The outcome was modeled using both linear and logistic regression. In the first case, mRS was treated as a numerical variable, and, in the second case, the dichotomized mRS (favorable vs. unfavorable) was used. cvMTT was used as a predictor in both cases. In addition, logistic regression based on the logarithm of cvMTT was performed. Both logistic regressions yielded essentially the same results. Subgroup analysis was performed to evaluate a possible influence of intracranial pressure or anesthesia. A distinction was made between the patients without EVD and the patients in an anesthetized state with an EVD at the time of CTP. One outlier was excluded from the analysis of the correlation between the cvMTT and the mRS. This outlier, with an mRS of 6, showed a cvMTT that was almost three times higher than the highest value of the rest of the study population, which cannot be logically explained, and is most likely due to mismeasurement. All calculations were performed in R, version 4.1.1.3.

## 3. Results

### 3.1. Patient Cohort

During the study period, 476 patients presented with SAH on an initial nonenhanced CT scan. After applying our inclusion criteria, 136 patients had CTP scans within 24 h after admission, with good image quality, and a documented outcome at 6 months. In 31 patients, two CTP scans were performed within the first 24 h after admission, but only the first was included (a total of 167 CTP in 136 patients). However, in 11 patients who were excluded, the ictus could not be determined with certainty or preceded the CTP by more than 24 (remaining: 125 patients). In one patient, the aneurysm had already been treated at the time of the first CTP scan, resulting in exclusion from the analysis. A total of 124 patients remained for analysis in this study.

A total of 83 patients of the study cohort (67%) were female, 41 patients (33%) were younger than 50 years, and 83 patients (67%) were aged 50 years or older. On admission, 61 patients (49%) had a good neurologic grade (WFNS I–III), and 63 patients (51%) had a poor grade aSAH (WFNS IV–V). A total of 21 patients (18%) had mild aSAH (modified Fisher grade 1–2), and 102 (82%) had severe aSAH (modified Fisher grade 3–4). At the time of early imaging, 79 patients (64%) already had an EVD in place, and 45 (36%) did not have an EVD. Table 1 shows the patient characteristics for these two groups in detail. In total, 59 patients (48%) received endovascular aneurysm treatment, 59 patients (48%) were treated surgically, 2 patients (1%) received neither treatment, and 4 patients (3%) received both endovascular and surgical treatment.

Within the first 6 months after ictus, 24 patients (19.3%) died (mRS 6), another 24 patients (19.3%) had an unfavorable outcome (mRS 3–5), and 76 patients (61.3%) were in a favorable clinical condition (mRS 0–2) (Table 1).

### 3.2. Early MTT Heterogeneity

**Figure 1:**
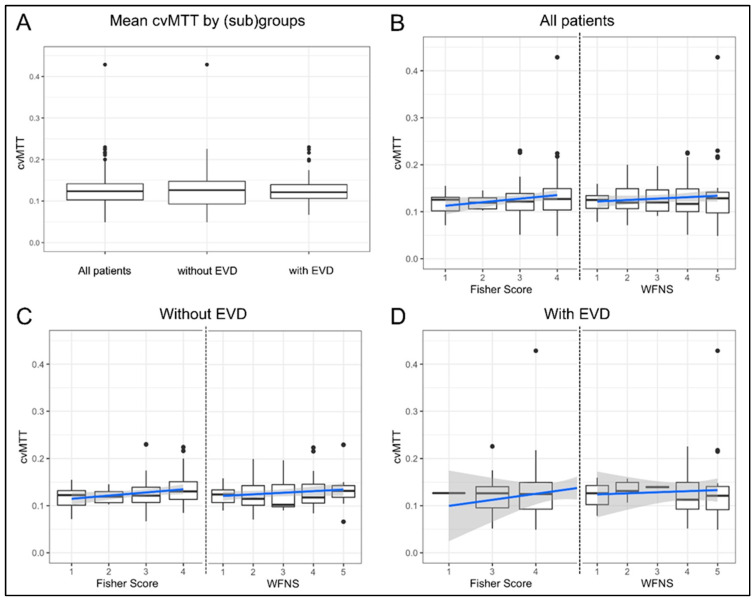
Mean cvMTT and correlation of cvMTT with Fisher and WFNS grades (boxplot with overlaid linear regression). (**A**) There is no significant difference between the mean cvMTT for all patients (*n* = 124) and the subgroups of patients with (*n* = 79) and without EVD (*n* = 45) at the time of CTP imaging. (**B**) cvMTT distribution of the Fisher grade (**left**) and the WFNS grades (**right**) for all patients. The cvMTT did not correlate with the initial WFNS grade (*p* = 0.23) or Fisher grade (*p* = 0.07) at admission. (**C**) cvMTT distribution of the Fisher grade (**left**) and the WFNS grades (**right**) in the subgroup of patients without EVD. The cvMTT did not correlate with the initial WFNS grade (*p* = 0.15) but correlated weakly with the Fisher grade (*p* = 0.05) at admission. (**D**) cvMTT distribution of the Fisher grade (**left**) and the WFNS grades (**right**) in the subgroup of patients with EVD. The cvMTT did not correlate with the initial WFNS grade (*p* = 0.76) or Fisher grade (*p* = 0.39) at admission. All figures: black dots = outliers; blue = regression line; grey = confidence band. The dotted line separates the two scales, Fisher score and WFNS grade.

The mean cvMTT of early perfusion imaging in all of the patients was 0.13 ± 0.05. In the subgroup of patients with and without a prior EVD placement, the mean cvMTT was 0.13 in each case (0.13 ± 0.03 for the patients without EVD and 0.131 ± 0.0613 for those with EVD; Figure 1A, *p* = 0.69).

The cvMTT did not significantly correlate with the WFNS grade in the whole study population (β = 0; 95% CI, 0–0.01; *p* = 0.23), or in the subgroup with and without EVD (β = 0; 95% CI, −0.01–0.02; *p* = 0.76 and β = 0; 95% CI, 0–0.01; *p* = 0.15, respectively; Figure 1B–D). Again, there was no correlation between the cvMTT and the amount of subarachnoid blood, as assessed by the Fisher grade in the entire study population (β = 0.01; 95% CI, 0–0.02; *p* = 0.07) or in the subgroup with EVD (β = 0.01; 95% CI, −0.02–0.04; *p* = 0.39). However, there is a weak positive correlation between the cvMTT and the Fisher grade in the subgroup of patients without EVD (β = 0.01; 95% CI, 0–0.01; *p* = 0.05; Figure 1B–D).

### 3.3. Early CTP Heterogeneity Does Not Correlate with 6-Month mRS

**Figure 2:**
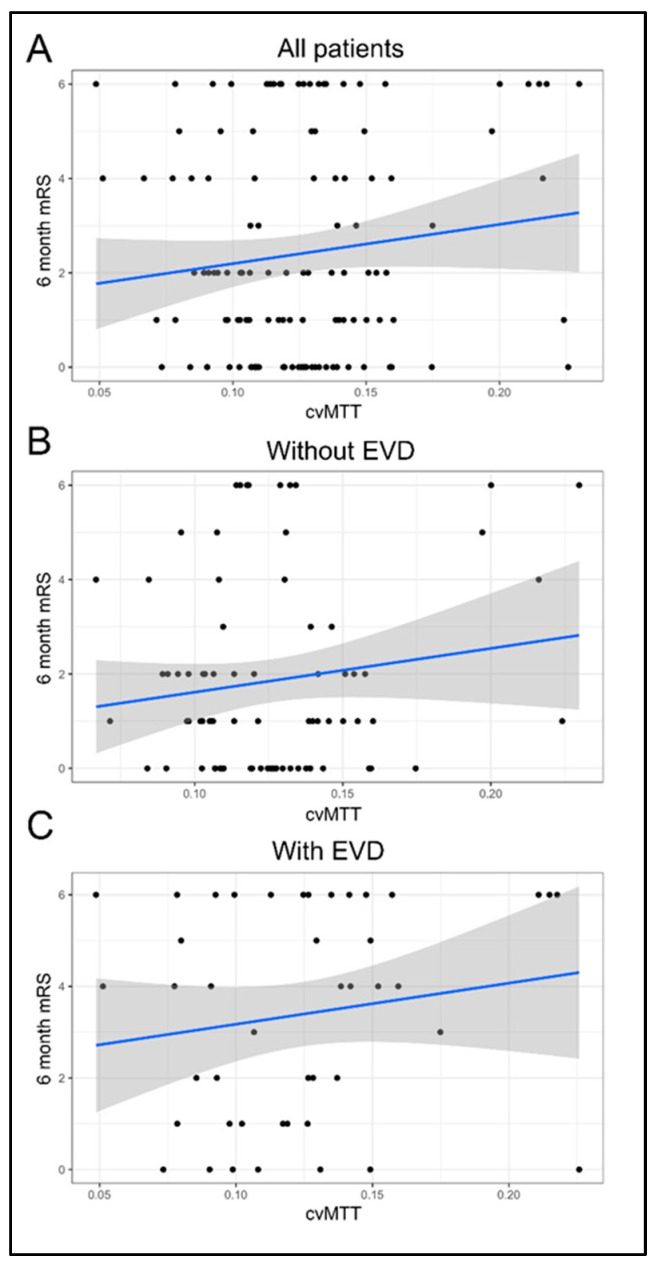
Correlation of cvMTT with the outcome after 6 months. (**A**) cvMTT as a predictor of the mRS after 6 months for all patients (linear regression: β = 8.3, 95% CI = (−3, 19.6); *p* = 0.15). (**B**) cvMTT as a predictor of the mRS after 6 months in the subgroup of patients without EVD (linear regression: β = 9.3, 95% CI = (−5.1, 23.7); *p* = 0.21). (**C**) cvMTT as a predictor of the mRS after 6 months in the subgroup of patients with EVD (linear regression: β = 9, 95% CI = (−7.7, 25.6); *p* = 0.3). All figures: black = empirical data; blue = regression line; grey = confidence band.

The cvMTT of early pCT did not significantly correlate with the mRS score after 6 months for the whole study population and both subpopulations (β = 8.32; 95% CI, −3–19.64; *p* = 0.16; without EVD: β = 9.28; 95% CI, −5.09–23.66; *p* = 0.21; with: β = 8.97; 95% CI, −7.65–25.59; *p* = 0.30; Figure 2).

## 4. Discussion

This evaluation of data on aSAH patients showed the following three main results:

(1) The heterogeneity of microvascular perfusion, as defined by cvMTT in early CTP imaging during the EBI phase of aSAH, did not significantly correlate with the dichotomized outcome after 6 months;
(2) There was no significant difference in the mean heterogeneity of microvascular perfusion, as defined by mean cvMTT, between patients with and without EVD at the time of early CTP imaging;
(3) The heterogeneity of microvascular perfusion, as defined by cvMTT at early CTP imaging, did not significantly correlate with the initial WFNS grade or Fisher score.

The present analysis suggests that the MTT heterogeneity in the phase of EBI after aSAH is neither an independent predictor for a poor outcome nor relates to the WFNS or Fisher grades. These observations from the EBI phase contrast with those from the DCI phase, where the MTT heterogeneity serves as a predictor of the outcome [25]. The differences between the EBI and DCI phases may be attributed to the distinct pathomechanisms underlying these phases.

DCI is one of the essential pathophysiological concepts of aSAH and involves a plethora of pathophysiological mechanisms, including macro vasospasms, microvascular dysfunction, changes in cortical signaling, energy depletion, calcium channel dysfunction, and more. Additionally, Østergaard and colleagues proposed the concept of high capillary transit time heterogeneity (CTH) as another mechanism contributing to DCI [21,22]. Besides cerebral blood flow (CBF), the CTH is considered to be a determining factor for the local oxygenation of the brain parenchyma [22]. Increased CTH leads to an increasing shunt of oxygenated blood through the capillary bed, resulting in a dramatic deterioration of oxygenation in the surrounding brain tissue.

Recently, we demonstrated, for the first time, high DCI-related heterogeneity of cerebral microvascular perfusion clinically. High heterogeneity of MTT (cvMTT), as assessed by CTP scanning, was an independent predictor of a poor outcome in patients with aSAH [25]. The correlation of cvMTT is well explained by various local pathomechanisms that influence the microvascular perfusion of the brain parenchyma during the DCI phase, resulting in a high heterogeneity of MTT.

However, MTT heterogeneity may not directly reflect CTH, due to insufficient spatial resolution. Yet, by using cortical ROI bands with a spacing of 2° in the measurement of the heterogeneity of MTT, it clearly represents micro, rather than macro, perfusion heterogeneity, which can be assigned to specific vascular territories. Ultimately, the heterogeneity of the MTT can be considered a measure of the severity of DCI and, therefore, correlates with the outcome [25].

**Figure 3:**
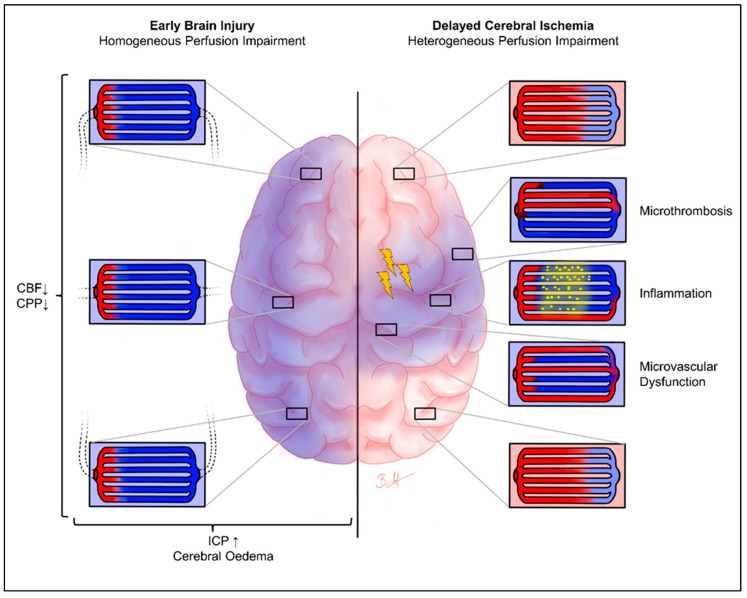
Heterogeneity of cerebral perfusion: EBI vs. DCI. Chematical representation of brain oxygenation in the phase of EBI (**left**) with homogeneous microvascular perfusion impairment, as well as DCI (**right**) with heterogeneous microvascular perfusion impairment. The blue staining shows potentially hypoxic areas, and the red coloring shows regular oxygenation. The enlarged images show the pathological processes that may be present in the respective areas. In the EBI phase, there is a uniformly distributed hypoxia of the entire brain parenchyma. In contrast, the DCI phase shows a heterogeneous picture of the microvascular perfusion of the brain parenchyma, due to various pathomechanisms (only chemically indicated here). The yellow flashes symbolize possible cortical spreading depolarization/depression. Arrows pointing upwards symbolise an increase, arrows pointing downwards symbolise a decrease. CBF, cerebral blood flow; CPP, cerebral perfusion pressure; ICP, intracranial pressure.

In contrast, EBI follows completely different pathomechanisms. Aneurysm rupture and subarachnoid hemorrhage lead to an increase in ICP, cerebral edema, and a steep increase in cerebral perfusion, resulting in (transient) global ischemia and the (complete) disruption of neuronal signaling [6]. Therefore, the pathomechanism of cerebral ischemia is entirely different from that of DCI. In DCI, cerebral hypoperfusion is related to focal macro vasospasms and microvascular dysfunction, thrombosis, etc., leading to focal ischemia. Moreover, various specific molecular mechanisms, such as calcium regulation, are induced [6,31]. In EBI, cerebral hypoperfusion is related to increased ICP, cerebral edema, cardiac output failure by neuro–cardiac coupling, etc., resulting in global hypoxia (Figure 3). Intracranial pressure approaches, or even exceeds, the mean arterial pressure for a short time, and a cerebral perfusion arrest can occur [10,11,32]. Therefore, we hypothesized that microvascular perfusion heterogeneity and capillary transit time heterogeneity are clinically not relevant in the phase of EBI. The result of the present study indicating that there is no correlation between the cvMTT of early CTP imaging and mRS after 6 months strengthens this hypothesis. It seems as if cvMTT, as a measure of the microvascular perfusion heterogeneity, may neither be clinically nor physiologically relevant in the EBI phase and, therefore, may not represent a predictor for the 6-month outcome following aSAH.

Another interesting finding of this study is that there is no significant difference in the absolute cvMTT values between the patients with and those without EVD in early CTP imaging. This indicates that cvMTT is most likely not significantly affected by intracranial pressure. We did not examine whether the intracranial pressure influences MTT itself, but intracranial pressure is believed to affect the time-to-peak/Tmax more than the MTT [20,33].

The results of this study on cvMTT in EBI, and the previous data on cvMTT in DCI, demonstrate an interesting additional parameter of CT perfusion imaging in the clinical setting. In our present analysis, we have gained further insight into the pathophysiology of EBI. Here, the inhomogeneity of microvascular perfusion seems to be less relevant than the impairment of general microvascular perfusion itself. Therefore, ensuring adequate cerebral perfusion at a very early stage should be an important therapeutic goal in the EBI phase. Supporting this evaluation, the relationship between cerebral microvascular perfusion and blood pressure was recently clinically demonstrated in the EBI phase [34]. In contrast, in DCI, not only the reduction in perfusion itself, but also the heterogeneity of microvascular perfusion, are pathophysiologically relevant. Future therapy concepts may aim to not only improve general microvascular perfusion, but also to achieve more homogeneous microvascular cerebral perfusion.

### Limitations

We acknowledge the following limitations of our study: (1) Even though the data were primarily collected prospectively, this is a retrospective evaluation of a new question. Not all of the patients received early imaging by CTP within 24 h of bleeding and prior to aneurysm repair, which is essential for this study. This may have led to a selection bias of the included patients, affecting this study’s results. However, early perfusion measurements are not established in many neurovascular centers. To our knowledge, we acquired one of the largest cohorts with regular early CTP imaging in aSAH patients. Therefore, we were able to include a considerable number of patients in the present monocentric study. Subsequently, good- and poor-grade aSAH cases are well balanced, making a selection bias less likely. However, the results of this study should be validated in a large prospective, preferably multicentric, study. (2) Again, our CTP setup is likely neither be able to measure capillary perfusion nor transit time heterogeneity, as described by Østergaard. CTP scanning was used in our study to measure the microvascular cerebral perfusion in a cortical band with ROIs 2° apart. The heterogeneity of microvascular perfusion between these ROIs, therefore, likely represents more micro and less macro perfusion, which can be assigned to specific vascular territories. A non-invasive measurement of cerebral capillary perfusion is difficult and has not yet been clinically established. (3) The effects of some potential confounders, such as additional macro vasospasms, comorbidities, intracerebral hemorrhage, brain edema, or seizures, were not analyzed for statistical reasons. A larger number of subgroups would severely limit the possibilities of statistical analysis, or even make it impossible. Diabetes, atherosclerosis, and hypertension may affect the microvasculature. However, these effects might be more relevant in DCI, with its high microvascular heterogeneity, rather than in EBI. (4) The established scores, such as the WFNS or Hunt and Hess scores, are clinically used to estimate the severity of aSAH and the patient’s outcome. Again, the early MTT and cvMTT in the phase of DCI are additional valuable predictive markers [7,8,15]. The use of advanced technologies such as machine learning to classify patients based on such established scores and microvascular perfusion markers is also conceivable; however, it is currently limited by the size of the current study population. Future prospective studies with a larger cohort might address these comorbidities and may enable a more detailed evaluation. (5) ICP measurements at the exact timepoint of CTP were not retrospectively available and, therefore, were not considered. ICP may affect cerebral perfusion. However, strict ICP management was performed on all of the patients with an EVD, and the patients without an EVD can be assumed to have normal ICP. Even if there was a difference in intracranial pressure between the patients with and those without EVD, the lack of a difference in cvMTT in the two subgroups suggests a negligible effect of ICP on cvMTT. However, in further prospective studies, ICP measurements should be performed at the time of CTP acquisition. (6) The only outcome parameter that was used in this study was the six-month mRS. Further studies might also include a longer follow-up and other outcome measures, e.g., psycho-cognitive outcome assessments. (7) Between different neurovascular centers, CTP imaging protocols and post-processing often differ significantly, therefore, our results may not be directly transferable to other setups. (8) Standard values for microvascular perfusion heterogeneity in healthy individuals have not yet been established. (9) A desirable direct comparison of microvascular perfusion heterogeneity between the EBI and DCI phases might give more insights into the potential pathophysiological processes and a potential pathophysiological connection between EBI and DCI. However, such a direct comparison is not possible, as the EBI patients in our study represent a distinct cohort from the DCI evaluation that was previously published. Future studies should aim to enable this direct comparison in a larger study population. (10) In the present work, we performed an evaluation of supratentorial perfusion, independent of the location of the bleeding source. Infratentorial perfusion and the possible associated inhomogeneities were not examined. We cannot exclude the possibility that infratentorial microvascular perfusion inhomogeneities may be pathophysiologically relevant to EBI. However, there is no indication of such a possible association in the literature to date.

## 5. Conclusions

In the present cohort of 124 patients with aSAH, the heterogeneity of MTT (cvMTT) of early cerebral perfusion imaging did not correlate with the initial WFNS grade, the Fisher grade, or the outcome after six months. Therefore, the present study suggests that microvascular perfusion heterogeneity, as measured by cvMTT, may not be clinically or physiologically relevant in the early brain injury (EBI) phase and, therefore, does not represent a predictor for the 6-month outcome following aSAH.

## Figures and Tables

**Table 1:** **Patient characteristics.** The percentages given refer to the total number of patients per subgroup. ACA, anterior cerebral artery; Acom, anterior communicating artery; BA, basilar artery; ICA, internal carotid artery; MCA, middle cerebral artery; mRS, modified Rankin scale; N, number of patients; PcaA, pericallosal artery; PCOM, posterior communicating artery; PICA, posterior inferior cerebellar artery; SCA, superior cerebellar artery; VA, vertebral artery.

	All Patients	With EVD	Without EVD
	No. (%)	No. (%)	No. (%)
Total	124 (100)	45 (100)	79 (100)
Age			
<50 years	41 (33.1)	8 (17.8)	33 (41.8)
≥50 years	83 (66.9)	37 (82.2)	46 (58.2)
Sex			
Female	83 (66.9)	32 (71.1)	51 (64.6)
Male	41 (33.1)	13 (28.9)	28 (35.4)
WFNS grade			
I–III	61 (49.2)	10 (22.2)	51 (64.6)
IV–V	63 (50.8)	35 (77.8)	28 (35.4)
Fisher grade			
0–II	22 (17.7)	1 (2.2)	21 (26.6)
III–IV	102 (82.3)	44 (97.8)	58 (73.4)
Aneurysm location			
ACA	1 (0.8)	0 (0.0)	1 (1.3)
Acom	49 (39.5)	17 (37.8)	32 (40.5)
BA	12 (9.7)	8 (17.8)	4 (5.1)
ICA	5 (4.0)	3 (6.7)	2 (2.5)
MCA	21 (16.9)	6 (13.3)	15 (19.0)
PcaA	4 (3.2)	1 (2.2)	3 (3.8)
PCOM	14 (11.3)	2 (4.4)	12 (15.2)
PICA	10 (8.1)	7 (15.6)	3 (3.8)
SCA	2 (1.6)	0 (0.0)	2 (2.5)
VA	4 (3.2)	1 (2.2)	3 (3.8)
Other	2 (1.6)	0 (0.0)	2 (2.5)
Therapy			
Endovascular	59 (47.6)	24 (53.3)	35 (44.3)
Surgical	59 (47.6)	19 (42.3)	40 (50.6)
Combined/no treatment	6 (4.8)	2 (4.4)	4 (5.1)
mRS 6 months			
0–2	76 (61.3)	18 (40.0)	58 (73.4)
3–5	24 (19.4)	12 (26.7)	12 (15.2)
6	24 (19.4)	15 (33.3)	9 (11.4)

## Data Availability

The datasets used and/or analyzed during the current study are available from the corresponding author upon reasonable request.

## Supplementary Materials

The following supporting information can be downloaded at: https://www.mdpi.com/article/10.3390/brainsci13050824/s1. Supplementary Figure S1—Schematic representation of the early CTP image workflow. (A) A series of axial CTP scans positioned at the level of the central parts of lateral ventricles, parallel to a plane through the orbital floor and the external auditory meatus, thereby sampling the anterior, middle, and posterior cerebral artery territories, as well as the anterior and posterior border zones. (B) The calculation of the parameter images (MTT, CBF, CBV, and Tmax) was performed using STROKETOOL-CT (Digital Image Solutions, Frechen, Germany), which processes data using singular value decomposition. (C) Depiction of the ROI automatically defined along the cortex. The readout starts in the territory of the right posterior cerebral artery and is carried out in a clockwise manner using a sliding-window approach (mean across 10° in 2° steps). (D) Representation as a histogram of 180 measurements across 360° (red, MTT; black, Tmax; yellow, rCBF; white, rCBV). (E) Example of the 180 individual values in numerical form, displayed from angle 0 to 10. (F) Calculation of the cvMTT from the 180 individual values. Adopted in modified form from Hofmann BB, Fischer I, Engel A, et al. MTT Heterogeneity in Perfusion CT Imaging as a Predictor of Outcome after Aneurysmal SAH. AJNR Am J Neuroradiol. 2021;42(8):1387-1395. doi:10.3174/ajnr.A7169 [25].

## Abbreviations

aSAH, aneurysmal subarachnoid hemorrhage; CBF, cerebral blood flow; CBV, cerebral blood volume; CI, confidence interval; CTH, capillary transit time heterogeneity; CTP, computed tomography perfusion; CT, computed tomography; cvMTT, coefficient of variation for mean transit time; DCI, delayed cerebral ischemia; EBI, early brain injury; EVD, external ventricular drainage; ICP, intracranial pressure; MTT, mean transit time; mRS, modified Rankin scale; ROI, region of interest; Tmax, time to maximum; WFNS score, World Federation of Neurological Surgeons score.
