# Supplementary material for: Evaluation of MTT Heterogeneity of Perfusion CT Imaging in the Early Brain Injury Phase: An Insight into aSAH Pathopysiology
Source: Brain Sci. 2023 May 19;13(5):824. doi: 10.3390/brainsci13050824 (PMC10216289; doi:10.3390/brainsci13050824)
Supplement: Supplementary file 1 [file brainsci-13-00824-s001.zip › brainsci-2344360-supplementary.pdf]

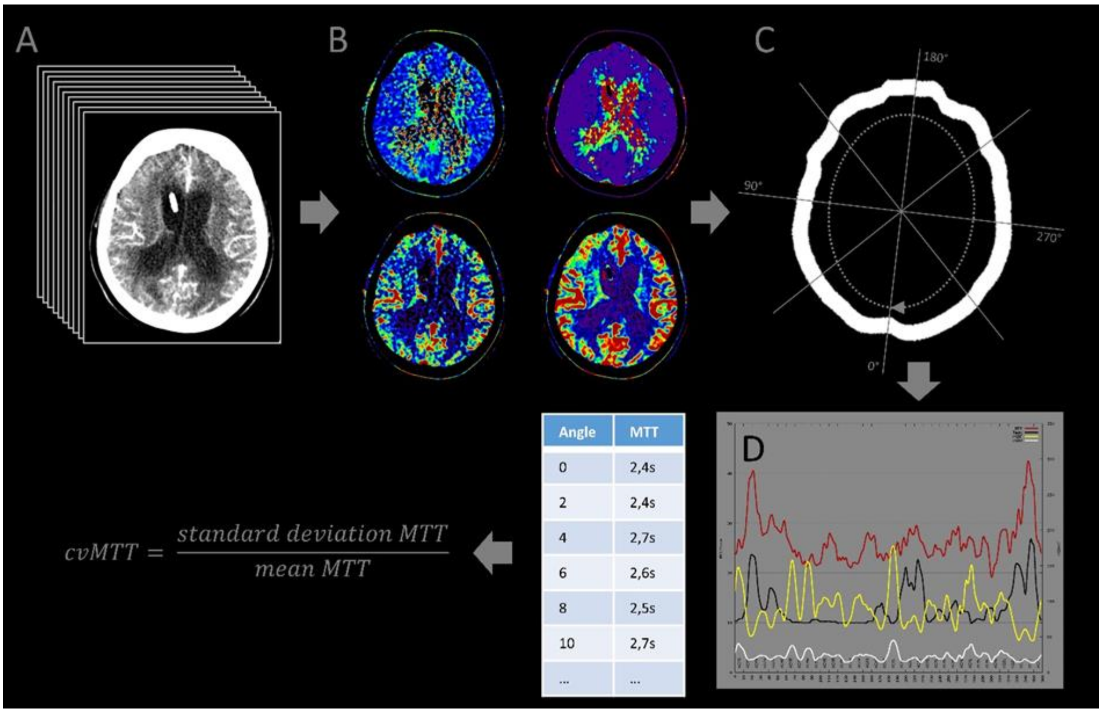

Supplementary Figure S1 - Schematic representation of the early CTP image workflow. A, Series of axial CTP scans positioned at the level of the central parts of lateral ventricles, parallel to a plane Table 10. in 2° steps). D, Representation as histogram of 180 measurements across 360° (red, MTT; black, Tmax; yellow, rCBF; white, rCBV). E, Example of the 180 individual values in numerical form, displayed from angle 0 to 10. F, Calculation of the cvMTT from the 180 individual values. *Adopted in modified form from Hofmann BB, Fischer I, Engel A, et al. MTT Heterogeneity in Perfusion CT Imaging as a Predictor of Outcome after Aneurysmal SAH. AJNR Am J Neuroradiol. 2021;42(8):1387-1395. doi:10.3174/ajnr.A7169.*
